# *Brunneosporopsis yunnanensis* gen. et sp. nov. and *Allocryptovalsa xishuangbanica* sp. nov., New Terrestrial Sordariomycetes from Southwest China

**DOI:** 10.3390/life12050635

**Published:** 2022-04-25

**Authors:** Sajeewa S. N. Maharachchikumbura, Dhanushka N. Wanasinghe, Abdallah M. Elgorban, Salim S. Al-Rejaie, Elham A. Kazerooni, Ratchadawan Cheewangkoon

**Affiliations:** 1 Center for Informational Biology, School of Life Science and Technology, University of Electronic Science and Technology of China, Chengdu 611731, China; sajeewa83@yahoo.com or; 2 Department of Entomology and Plant Pathology, Faculty of Agriculture, Chiang Mai University, Chiang Mai 50200, Thailand; 3 Honghe Center for Mountain Futures, Kunming Institute of Botany, Chinese Academy of Sciences, Honghe County 654400, China; 4 Department of Botany and Microbiology, College of Science, King Saud University, Riyadh 11451, Saudi Arabia; aelgorban@ksu.edu.sa; 5 Department of Pharmacology and Toxicology, College of Pharmacy, King Saud University, Riyadh 11451, Saudi Arabia; rajaie@ksu.edu.sa; 6 School of Applied Biosciences, Kyungpook National University, Daegu 41566, South Korea; elham.ghasemi.k@gmail.com

**Keywords:** Diaporthomycetidae, Greater Mekong Subregion, Hypocreales, microfungi, Sichuan, Xylariomycetidae, Yunnan

## Abstract

Three fungal taxa were collected on dead branches of wood during fieldwork in Sichuan and Yunnan Provinces, China. The new generic name *Brunneosporopsis* gen. nov. and species *B*. *yunnanensis* sp. nov. are introduced for a novel taxon characterized by globose to subglobose and dark olivacous-brown conidia. Phylogenetic analyses based on combined LSU, SSU and *tef*1-α loci strongly support the monophyly of this taxon and place it in the subclass Diaporthomycetidae. It could not be assigned to any currently recognized families in the subclass and was, therefore, placed in the Diaporthomycetidae genera *incertae sedis*. A second taxon represents a new species in *Allocryptovalsa* based on an analysis of the sequence datasets of ITS and *btub* loci of the novel, brown-spored sexual morphic species. This taxon is described here as *A*. *xishuangbanica* sp. nov. An interesting hypocrealean fungus producing synnemata, *Stilbocrea gracilipes*, was collected from dead wood of an unknown host from Sichuan Province and is reported here, with asexual morph from both the host and culture as well as LSU, ITS, *tef*1-α, *rpb*2 and *rpb*1 sequence data.

## 1. Introduction

Sordariomycetes is a large and taxonomically complex group of asexual and sexual ascomycetes that are common throughout the world, including China, occupying various ecological niches [1,2,3,4,5]. Many species of Sordariomycetes are pathogens of important crops [6]. Sordariomycetes are also frequently isolated as endophytes from numerous plants [7]. Some taxa are fungicolous, while many persist as saprobes involved in decomposition and nutrient-recycling [6,8]. Some species of Sordariomycetes are economically important biocontrol agents, and others produce a wide range of chemically diverse metabolites important in agricultural, medicinal and other biotechnological industries [9,10,11,12].

The class is characterized by taxa with perithecial ascomata, paraphysate hamathecium, periphysate ostioles and unitunicate or pseudoprotunicate asci [4,6]. Based on ribosomal RNA sequence data, combined with morphology, Eriksson & Winka [13] defined three subclasses: Hypocreomycetidae, Sordariomycetidae and Xylariomycetidae. Using morphological characteristics, ecological data and analyses of combined sequence data (LSU, SSU, *tef*1-α and *rpb*2), Maharachchikumbura et al. [8] introduced three other subclasses: Diaporthomycetidae, Lulworthiomycetidae and Meliolomycetidae. However, the subclass Meliolomycetidae is considered a synonym of Sordariomycetidae [14], while Savoryellomycetidae was introduced in a later study [4]. The class Sordariomycetes presently consists of six subclasses [15].

We are carrying out an inventory of the species composition of pathogenic and saprobic Sordariomycetes in Southwest China and establishing their taxonomic positions using sequence data. In the present study, we isolated three interesting saprobic fungi from dead wood in Sichuan and Yunnan Provinces. Based on initial microscopic investigations of the taxa, the organisms were identified as fungi belonging to Sordariomycetes. We reveal that two of these taxa are new to science and describe them here based on multigene sequence data.

## 2. Materials and Methods

### 2.1. Herbarium Material

Fresh fungal material was collected from dead woody sticks of deciduous hosts from Chengdu (UTM/WGS84: 48R 103.924170° E, 30.757546° N, 533 m above sea level, in the rainy season, August 2021), Qujing (UTM/WGS84: 48R 104.149958° E, 2896195.66° N, 1845 m above sea level, in the rainy season, May 2019) and Xishuangbanna (UTM/WGS84: 47Q, 101.29861111° E, 21.90000028° N, 590 m above sea level, during the dry season, November 2020) in Yunnan Province, China. Dry herbarium materials were deposited in the herbarium of Cryptogams Kunming Institute of Botany, Academia Sinica (KUN-HKAS), and MycoBank numbers were registered as outlined by MycoBank (http://www.MycoBank.org, accessed on 21 March 2022).

### 2.2. Morphological Observations

Morphological external and internal macro-/micro-structures were noted per Wanasinghe et al. [16]. Macro- and micromorphological features were photographed using an Olympus SZ61 Series and a Nikon ECLIPSE Ni (Nikon Instruments Inc., Melville, NY, USA) compound microscope. Macroscopic images of colonies were documented using an iPhone XS Max (Apple Inc., Cupertino, CA, USA) with daylight. At least 20 measurements were taken for each structure using the Tarosoft (R) Image Frame Work program. All photographs were arranged using Adobe Photoshop CS6 (Adobe Systems, San Jose, CA, USA).

### 2.3. DNA Isolation, PCR Amplification and Sequencing

Genomic DNA was extracted from the samples as described by Wanasinghe et al. [17] using the Biospin Fungus Genomic DNA Extraction KitBSC14S1 (BioFlux, Shanghai, China) following instructions of the manufacturer. Polymerase chain reaction (PCR) was performed with an automated thermal cycling device. For the PCR, the reference DNA was stored at 4 °C for regular use and duplicated at −20 °C for long-term storage. The internal transcribed spacer (ITS), 28S large subunit ribosomal ribonucleic acid (LSU), β-tubulin (*btub*), translation elongation factor 1-α (*tef*1-α), RNA polymerase II subunit 1 (*rpb1*) and RNA polymerase II subunit 2 (*rpb2*) loci were amplified by PCR reaction as described in Wanasinghe et al. [18] and Voglmayr & Jaklitsch [19]. The primers ITS5/ ITS4 were used for ITS [20], LR0R/LR5 for LSU [21], TUB2Fw/TUB4Rd for *btub* [22], EF1 983F/EF1-2218R for *tef*1-α [23,24], fRPB2-5F/fRPB2-7cR for *rpb2* [25] and RPB1-Ac/RPB1Cr for *rpb1* [26,27]. The successfully amplified PCR products with subsequent sequencing in both directions were outsourced to a commercial sequencing company (BGI, Ltd., Shenzhen, China).

### 2.4. Sequence Alignment and Phylogenetic Analysis

Raw sequence reads were assembled and edited using SeqMan Pro v. 8.1.3 (DNASTAR Lasergene, Madison, WI, USA) and stored at GenBank under accession numbers indicated in in the notes below. Initial BLAST results showed that the isolates belonged to *Allocryptovalsa*, *Stilbocrea* and Diaporthomycetidae. Hence, three different datasets were used to estimate three phylogenies: the *Allocryptovalsa* tree was based on combined ITS + *btub* regions, the *Stilbocrea* tree on combined LSU + ITS + *rpb*2 + *tef*1-α + *rpb*1 and the Diaporthomycetidae tree on a combined LSU + SSU + *tef*1-α dataset. DNA sequences were altered for each locus separately using MEGA v.7.0.26 [28] and adjusted manually. Phylogenetic analyses were conducted using maximum likelihood (ML) and Bayesian inference (BI). ML inference was performed on the CIPRES Science Gateway portal [29] as detailed in Maharachchikumbura et al. [30]. The BI suitable models were first selected using nucleotide substitution models for each gene as determined with MrModeltest v. 2.2 [31] and used for each gene partition. The BI (MrBayes v. 3.2.1; [31]) was conducted per Wanasinghe et al. [16]. Resulting trees were visualized with FigTree v. 1.4.4 (http://tree.bio.ed.ac.uk/software/figtree/, accessed on 11 March 2022), and the layout was completed with Adobe Illustrator® CS5 (Version 15.0.0, Adobe®, San Jose, CA, USA).

## 3. Results

### 3.1. Stilbocrea Analysis

**Figure 1:**
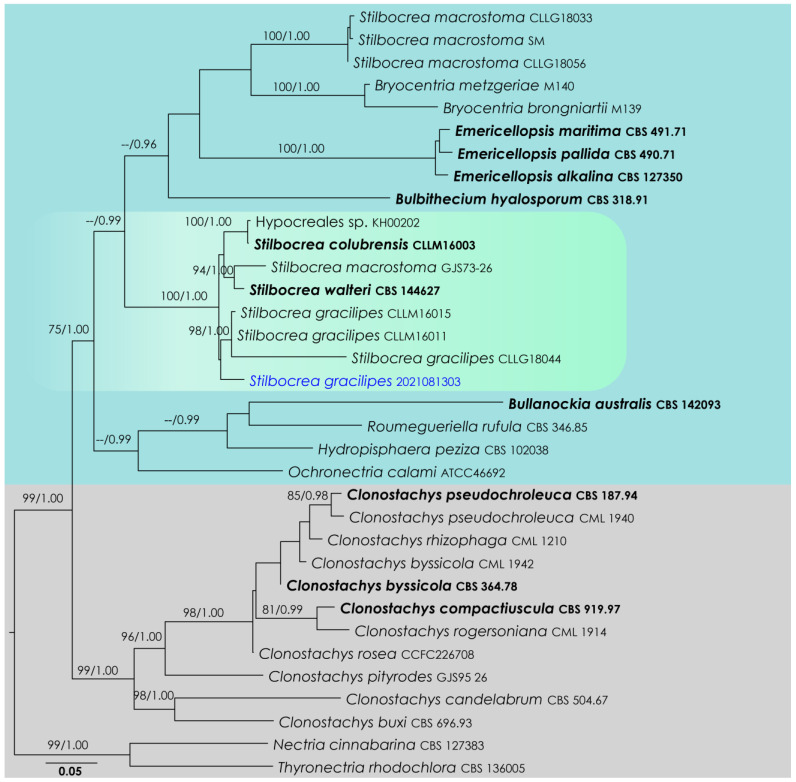
RAxML tree based on an integrated dataset of partial LSU, ITS, *rpb*2, *tef*1-α, *rpb*1 DNA sequence analysis in Hypocreales. Bootstrap support values for ML equal to or greater than 70% and BYPP equal to or greater than 0.95 are shown as ML/BI above the nodes. Blue represents new isolates. Species names given in bold indicate ex-type and ex-paratype strains. The scale bar represents the expected number of nucleotide substitutions per site.

*Stilbocrea* LSU, ITS, *rpb*2, *tef*1-α, *rpb*1 phylogeny (Figure 1): The alignment contained 28 isolates, and the tree was rooted to *Nectria cinnabarina* (CBS 127383) and *Thyronectria rhodochlora* (CBS 136005). The final alignment contained 4553 characters used for the phylogenetic analyses, including alignment gaps. The RAxML analysis of the integrated dataset generated a best-scoring tree with a final ML optimization likelihood value of −25,809.955191. The matrix featured 1908 distinct alignment patterns, with 55.67% undetermined characters or gaps. The parameters used for the GTR + I + G model of the amplicons were as follows: estimated base frequencies: A = 0.23939, C = 0.265158, G = 0.273786, T = 0.221666; substitution rates AC = 1.597464, AG = 2.879053, AT = 1.626395, CG = 1.175986, CT = 7.501803, GT = 1.000; proportion of invariable sites I = 0.454714; gamma distribution shape parameter α = 0.804034. Based on the results of MrModelTest, dirichlet base frequencies and the GTR + I + G model were used for the Bayesian analysis. The Bayesian analyses created 5601 trees (saved every 100 generations), out of which 4201 were sampled after 25% of the trees were discarded as burn-in. The alignment featured a total of 1909 unique site patterns. In the combined data analysis, the new isolate, SM-2021081303, nested in a clade comprising *Stilbocrea gracilipes* (CLLM16011, CLLM16015, CLLG18044). However, this relationship is not statistically supported (Figure 1).

### 3.2. Allocryptovalsa Analysis

**Figure 2:**
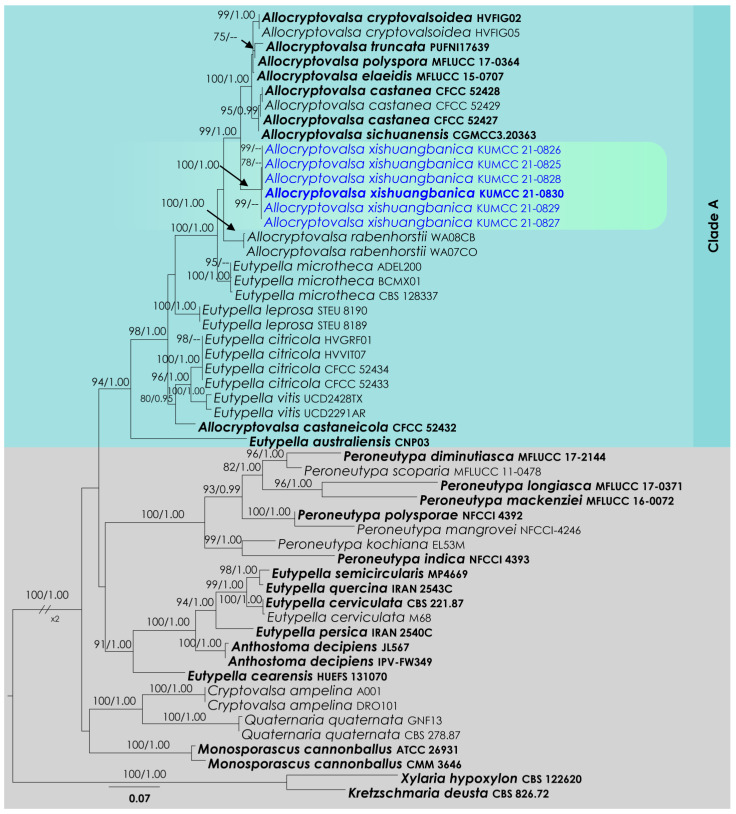
RAxML tree based on an integrated dataset of partial ITS and *btub* DNA sequence analysis in Diatrypaceae. Bootstrap support values for ML equal to or greater than 70% and BYPP equal to or greater than 0.95 are shown as ML/BI above the nodes. Blue represents new isolates. Species names given in bold indicate ex-type and ex-paratype strains. The scale bar represents the expected number of nucleotide substitutions per site.

*Allocryptovalsa* ITS and *btub* phylogeny (Figure 2): The alignment contained 54 isolates and the tree was rooted to *Kretzschmaria deusta* (CBS 826.72) and *Xylaria hypoxylon* (CBS 122620). The final alignment contained 1096 characters used for the phylogenetic analyses, including alignment gaps. The RAxML analysis of the combined dataset yielded a best-scoring tree with a final ML optimization likelihood value of −8779.183187. The matrix had 669 distinct alignment patterns, with 34.72% undetermined characters or gaps. The parameters for the GTR + I + G model of the combined amplicons were as follows: estimated base frequencies; A = 0.231173, C = 0.262694, G = 0.233192, T = 0.272942; substitution rates AC = 1.02372, AG = 2.846819, AT = 1.465774, CG = 0.902854, CT = 3.501434, GT = 1.000; proportion of invariable sites I = 0.337391; gamma distribution shape parameter α = 0.939536. Based on the results of MrModelTest, dirichlet base frequencies and the GTR + I + G model were used for the Bayesian analysis. The Bayesian analyses generated 2201 trees (saved every 100 generations), from which 1651 were sampled after 25% of the trees were discarded as burn-in. In total, the alignment featured 671 unique site patterns. In the combined data analysis, the six isolates described here as *Allocryptovalsa xishuangbanica*, in addition to *A. castaneae*, *A*. *castaneicola*, *A*. *cryptovalsoidea*, *A*. *elaeidis*, *A*. *polyspora*, *A*. *rabenhorstii*, *A*. *sichuanensis* and *A*. *truncata,* grouped as a monophyletic clade with 99% ML and 1.00 BI support (Figure 2).

### 3.3. Diaporthomycetidae Analysis

**Figure 3:**
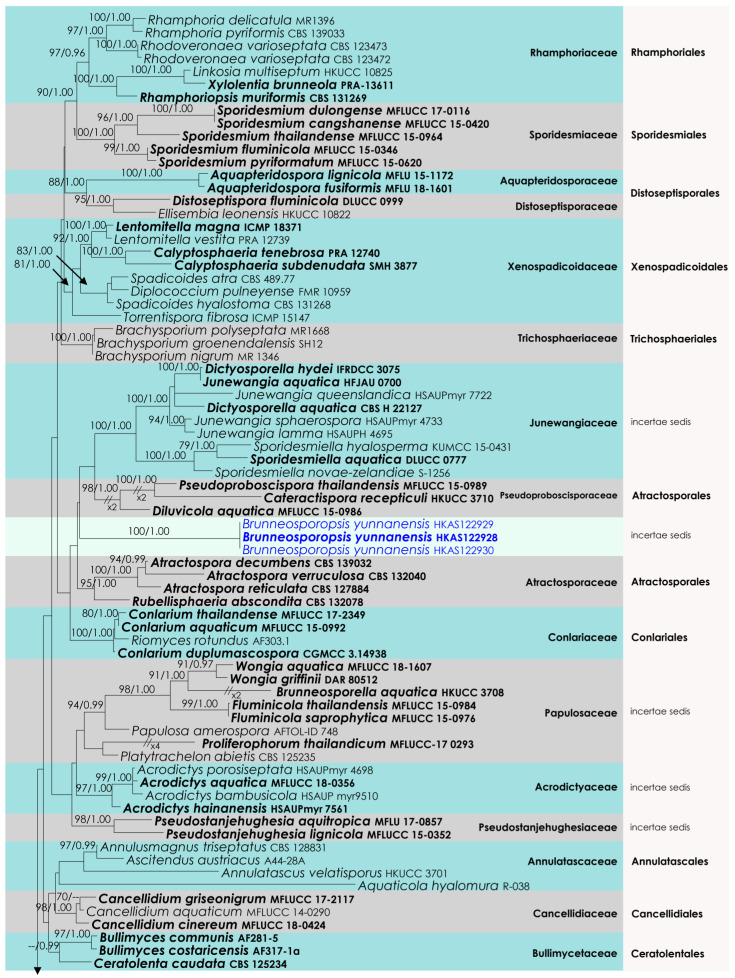

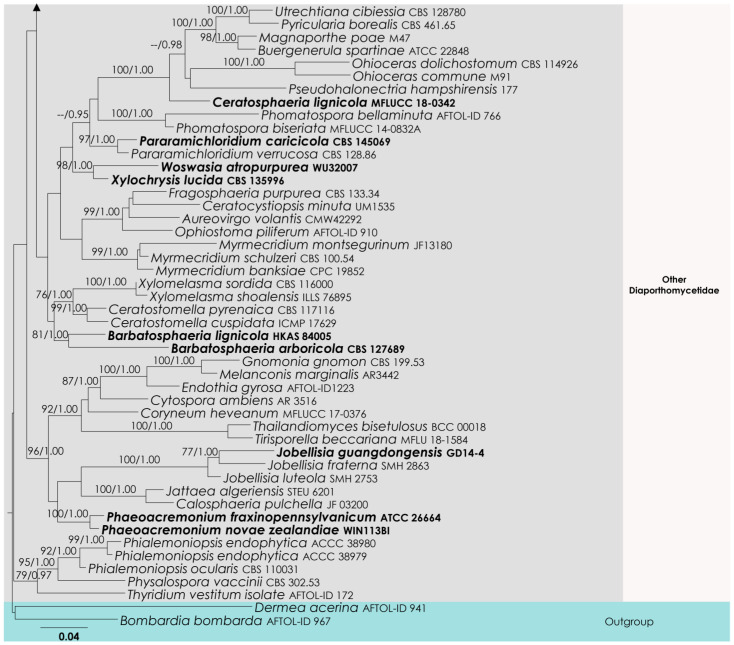
RAxML tree based on an integrated dataset of partial LSU + SSU + *tef*1-α DNA sequence analysis in Diaporthomycetidae. Bootstrap support values for ML equal to or greater than 50% and BYPP equal to or greater than 0.95 are shown as ML/BI above the nodes. Blue represents new isolates. Species names given in bold indicate ex-type and ex-paratype strains. The scale bar represents the expected number of nucleotide substitutions per site.

The aligned dataset included 121 isolates of Sordariomycetes, and the tree was rooted to *Dermea acerina* (AFTOL-ID 941) and *Bombardia bombarda* (AFTOL-ID 967). The final aligned dataset contained a total of 3522 characters (LSU = 1152, SSU = 1437, *tef*1-α = 933) used for the phylogenetic analyses, including alignment gaps. The RAxML analysis of the combined alignment generated the best scoring tree with a final ML optimization likelihood value of −36,700.143906 (Figure 3). All the rate heterogeneity models were estimated by the GTR best-fit model, with 1824 distinct alignment patterns and 45.28% undetermined characters or gaps. The estimated base frequencies were as follows: A = 0.249688, C = 0.237209, G = 0.283889 and T = 0.229214, with substitution rates AC = 0.839712, AG = 2.461384, AT = 1.106200, CG = 1.128735, CT = 6.420739 and GT = 1.000. The gamma distribution shape parameter alpha was 0.276590, and the tree-length = 6.039365. Our analysis generally agrees with Hyde et al. (2021). However, the family arrangement of Atractosporales does not agree with the previous studies [32,33,34]. The family Pseudoproboscisporaceae is more closely related to the Junewangiaceae, a family treated in Diaporthomycetidae family *incertae sedis*.

### 3.4. Taxonomy

**Hypocreomycetidae** O.E. Erikss. & Winka, Myconet 1: 6 (1997)

**Hypocreales** Lindau, Die Natürlichen Pflanzenfamilien nebst ihren Gattungen und wichtigeren Arten 1 (1): 343 (1897)

**Bionectriaceae** Samuels & Rossman (1999)

***Stilbocrea*** Pat., Bulletin de la Société Mycologique de France 16: 188, 186 (1900)

**Figure 4:**
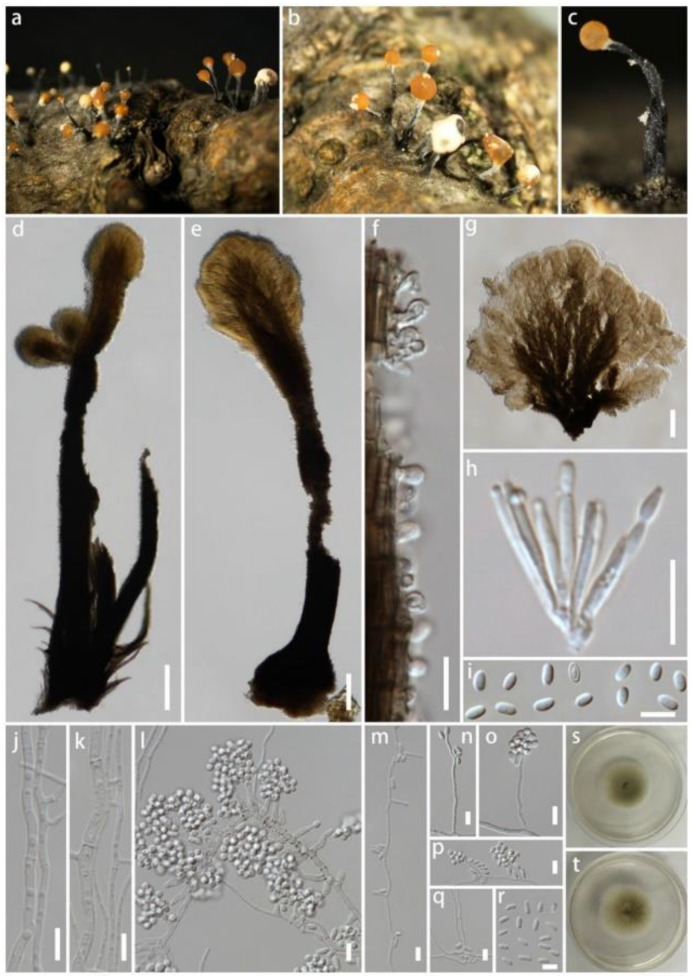
*Stilbocrea gracilipes* (H-2021081303). (**a**–**c**) Synnemata on substrate; (**d**–**f**) vertical section through the synnemata; (**g**) vertical section through the capitate apex; (**h**) phialides on natural substrate; (**i**) conidia on natural substrate; (**j**–**r**) conidiophores, phialides and conidia; (**s**,**t**) culture characteristic on PDA after 14 days. Scale bars: (**d**,**e**,**g**–**i**) 10 μm; (**f**,**j**–**r**) 5 μm.

***Stilbocrea gracilipes*** (Tul. & C. Tul.) Samuels & Seifert, in Rossman, Samuels, Rogerson & Lowen, Stud. Mycol. 42: 73 (1999) (Figure 4)

*Saprobic* on dead wood of unknown plant. Sexual morph: unobserved. Asexual morph: *synnemata* scattered, gregarious, erumpent through the bark, cylindrical-capitate, subulate-capitate to spatulate, straight, nodding or sinuous, slender, unbranched or branched, black, smooth to hirsute, 800–2000 μm tall, 50–220 μm wide in the middle, up to 350 μm wide at the base. *Hyphae* of stipe forming a *textura intricata* at base and *textura porrecta* in the stipe, black, frequently septate, with smooth, roughened or verrucose walls and hyphae less pigmented towards the apex. *Ornamenting cells* covering upper one third to three-quarters of stipe, globose, subglobose, ellipsoidal, clavate, obpyriform. *Conidiophore* branching once or twice. *Phialides* subulate or cylindrical, straight or curved, 8–20 μm long, 1–3 μm wide at base, proliferating percurrently, collarettes sometimes slightly flared and periclinal thickening present. *Conidial mass* shade of orange or brown, opaque or translucent, 50–500 μm diameter *Conidia* ellipsoidal, straight or slightly curved, hyaline, 4–7 × 2–3 μm, usually with two polar guttules and walls slightly thickened.

*Cultures* on PDA dull-green, slow-growing, 50–60 mm diameter after 16 days, plane, aerial mycelium absent and surface smooth with an entire margin. *Mononematous conidiophores* on PDA acremonium-like, with phialides 5–30 × 1–2 μm, arising from single hyphae or fascicles, conidia oblong-ellipsoidal 2–4 × 1.5–2.5 μm. *Pionnottes* forming with branched conidiophores, with mixtures of monochasial, biverticillate and two-level verticillate branching, phialides 10–50 × 1–1.5 μm. *Conidia* ellipsoidal, 1.5–3 × 1.5–3 μm.

Material examined: China, Sichuan Province, Chengdu, University of Electronic Science and Technology, on dead wood of unknown plant, 13 August 2021, Q. Li (H-2021081303), living culture SM-2021081303.

GenBank Numbers LSU: ON041116; ITS: ON041132; *rpb*2: ON081502, *tef*1-α: ON125559; *rpb*1: ON081503.

Notes: *Stilbocrea gracilipes* is common in pantropical, subtropical and temperate regions, growing on various substrates such as bark and wood of various plants [35]. *Stilbocrea gracilipes* is easily recognized by the combination of black synnemata and orange-color spore mass [36]. The phylogeny suggests that our new collection shares a closely related phylogenetic affinity with *S. gracilipes* isolates deposited by Lechat & Fournier [37]. Those isolates only have LSU and ITS sequences, and a base-pair comparison of our strain showed 0.6% (5/844) base-pair changes in LSU with 1.14% (6/527) base-pair changes in ITS. Further, it is notable that our collection forms relatively longer phialides resulting from pionnottes than the previous studies.

**Xylariomycetidae** O.E. Erikss. & Winka, Myconet 1: 12 (1997)

**Xylariales** Nannf., Nova Acta Regiae Societatis Scientiarum Upsaliensis 8 (2): 66 (1932)

**Diatrypaceae** Nitschke, Verh. Naturhist. Vereines Preuss. Rheinl.: 73 (1869)

***Allocryptovalsa*** Senwanna, Phookamsak & K.D. Hyde, Mycosphere 8 (10): 1839 (2017)

**Figure 5:**
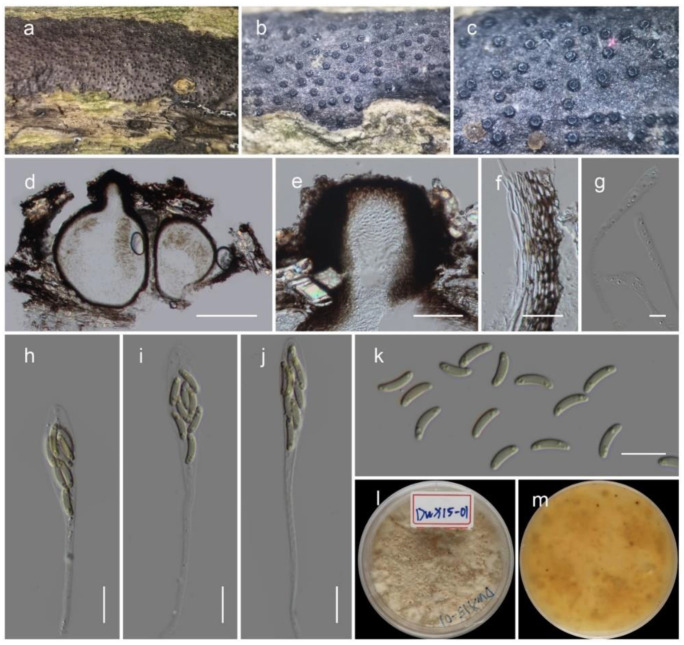
*Allocryptovalsa xishuangbanica* (HKAS122936, holotype). (**a**–**c**) Appearance of ascostromata on substrate; (**d**) vertical section through the ascoma; (**e**) ostiole with short periphyses; (**f**) peridium; (**g**) paraphyses; (**h**–**j**) asci; (**k**) ascospores; (**l**,**m**) culture characteristic on PDA after 14 days (m from below). Scale bars: (**d**) 200 μm; (**e**) 50 μm; (**f**) 20 μm; (**g**–**k**) 10 μm.

***Allocryptovalsa xishuangbanica*** Maharachch. & Wanas., sp. nov. (Figure 5)

MycoBank: MB 843438

Etymology: The specific epithet is derived from Xishuangbanna, Yunnan Province, China

*Saprobic* on dead branches of unknown host. Sexual morph: *stromata* solitary to gregarious, immersed to semi-immersed, erumpent through the surface of the bark. *Ectostromatic disc* brown, circular to oblong. *Perithecia* flask-shaped, perithecial necks erumpent in groups, 300–450 μm high, 250–350 μm diameter (M = 388 × 286 μm, *n* = 5). *Ostioles* numerous, gregarious, umbilicate, dark brown to black, 100–180 μm high, 40–60 μm diameter (M = 137 × 49 μm, *n* = 5). Peridium 15–25 μm wide, composed of two sections, outer layer dark brown, thick-walled cells, arranged in *Textura angularis*, inner layer hyaline, thin-walled cells of *textura prismatica*. *Hamathecium* composed of 2.5–4.5 μm-wide, filiform, septate, hyaline, unbranched paraphyses. *Asci* clavate to elongate obovoid, eight-spored, thin-walled, long pedicellate (25–35 μm, *n* = 30), apically flat, 60–80 long (including stalks), 7–10 μm diameter (M = 70 × 8.5 μm, *n* = 30). *Ascospores* elongate-allantoid, thin-walled, pale yellowish to pale brown at maturity, slightly curved, aseptate, 7–10.5 × 1.8–2.6 μm (M = 8.5 × 2.1 μm, *n* = 40). Asexual morph: not observed.

Culture characteristics: *colonies on PDA* reached a 2 cm diameter after 7 days at 20 °C. The colony was medium dense, irregular, flat or effuse, slightly raised, fluffy, white at the beginning and becoming cream-colored after 4 weeks with reverse cream-orange. The septate hyphae were branched, hyaline, thin and smooth-walled.

Material examined: China, Yunnan Province, Xishuangbanna, Mengla County, Menglunzhen, 21.90000028° N, 101.29861111° E, on a dead twig of an unknown deciduous host, 20 November 2020, D.N. Wanasinghe, DWX15-01-3 (HKAS122936, holotype), ex-type culture KUMCC 21-0830. *ibid*. DWX01-01-1 (HKAS122933), living culture KUMCC 21-0825, DWX15-01-1 (HKAS122935), living culture KUMCC 21-0829, DWX01-02-2 (HKAS122931), living culture KUMCC 21-0827, DWX01-01-4 (HKAS122932), living culture KUMCC 21-0826, DWX01-02-6 (HKAS122934), living culture KUMCC 21-0828.

GenBank Numbers LSU: ON041107–ON041112; ITS: ON041126–ON041131; SSU: ON041117–ON041122; *btub*: ON081496–ON081501; *tef*1-α: ON081504–ON081509.

Notes: *Allocryptovalsa xishuangbanica* (Figure 2 and Figure 5) is a morphologically and phylogenetically distinct species. All other species in *Allocryptovalsa* have polysporous asci, while *A. xishuangbanica* is the only species in the genus with eight-spored asci. *Allocryptovalsa rabenhorstii* (= *Cryptovalsa rabenhorstii*), which was isolated from *Vitis vinifera* in Australia and the United States [38], has close phylogenetic affinities to *A. xishuangbanica*.

**Diaporthomycetidae** Senan., Maharachch. & K.D. Hyde, Fungal Diversity 72: 208 (2015)

***Brunneosporopsis*** Maharachch. & Wanas., gen. nov.

MycoBank: MB 843435

Etymology: The generic epithet is from the word “brunius” (brown) for the color of the conidia.

*Saprobic* on dead branches of *Pinus yunnanensis*. Sexual morph: not observed. Asexual morph: *colonies* on natural substrate superficial, effuse, dark brown to black. *Conidiophores* micronematous, mononematous, erect, hyaline, smooth-walled often reduced to conidiogenous cells. *Conidiogenous cells* enteroblastic. *Conidia* solitary, globose to subglobose, or ovoid, dry, guttulate, dark brown to black when mature, smooth- to verruculose-walled.

Type: *Brunneosporopsis yunnanensis* Maharachch. & Wanas., sp. nov.

**Figure 6:**
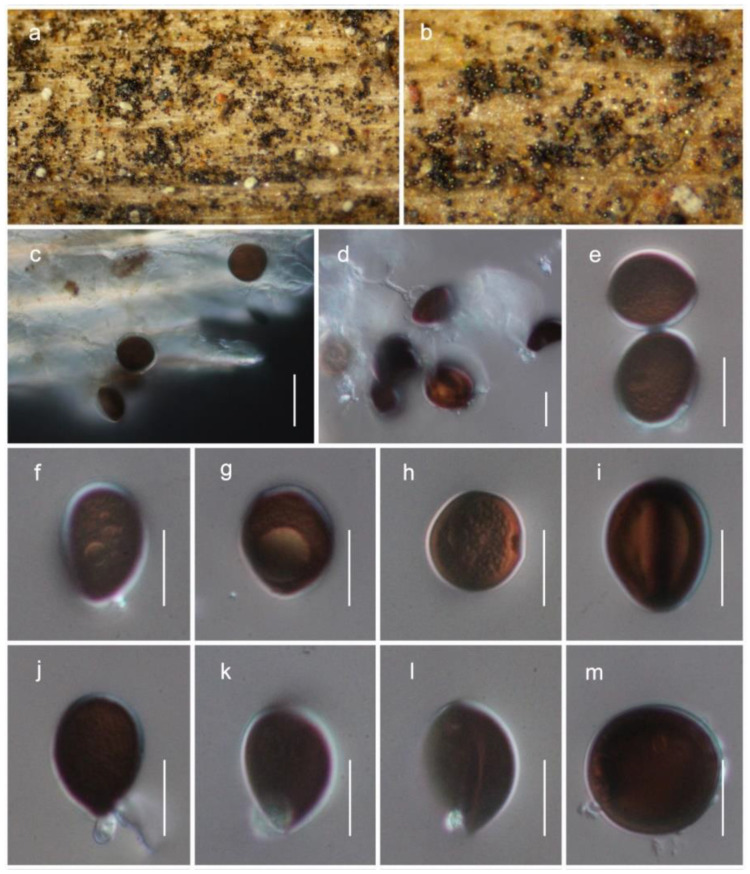
*Brunneosporopsis yunnanensis* (HKAS122928, holotype). (**a***–***c**) Conidia observed on host substrate; (**d**–**m**) conidia. Scale bars: (**c**) 20 μm; (**d**–**m**) 10 μm.

***Brunneosporopsis yunnanensis*** Maharachch. & Wanas., sp. nov. (Figure 6)

MycoBank: MB 843436

Etymology: The species was named after the province where the fungus was collected, Yunnan.

*Saprobic* on dead branches of *Pinus yunnanensis*. Sexual morph: not observed. Asexual morph: *colonies* on natural substrate superficial, effuse, dark brown to black. *Conidiophores* micronematous, mononematous, erect, hyaline, smooth-walled, often reduced to conidiogenous cells. Conidiogenous cells enteroblastic. *Conidia* solitary, 13−19 μm long, 10−17 μm wide (M = 15.5 ± 1.9 × 13.4 ± 2.1 μm, *n* = 25), globose to subglobose, or ovoid, dry, guttulate, dark brown to black when mature, smooth- to verruculose-walled.

Material examined: China, Yunnan Province, Qujing, Xuanwei, 26.182706 N, 104.149958 E, on dead branches of *Pinus yunnanensis* Franch. (Pinaceae), 15 May 2019, Li Huili, DW1799-02 (HKAS122928, holotype). *ibid*. 26.182805 N, 104.149957 E, DW1799-05 (HKAS122929), DW1799-06 (HKAS122930).

GenBank Numbers LSU: ON041113, ON041114, ON041115; SSU: ON041123, ON041124, ON041125; *tef*1-α: ON125559.

Notes: The new genus *Brunneosporopsis* forms a sister clade neighboring the families Junewangiaceae, Pseudoproboscisporaceae and Atractosporaceae, but support remains low (Figure 2). However, a significant difference in branch length suggests that *Brunneosporopsis* is distinct. *Brunneosporopsis* has arthrinium-like asexual morphs, which could not be seen in Junewangiaceae, Pseudoproboscisporaceae and Atractosporaceae. Due to phylogenetic and morphological distinctness, *Brunneosporopsis* could be raised to the level of family. However, we prefer to maintain this at the genus level until more cultures and collections become available. Furthermore, arthrinium-like morphology is common in other orders in Sordariomycetes, such as in Conioscyphales, Savoryellales and Xylariales, which are also phylogenetically distinct.

## 4. Discussion

Maharachchikumbura et al. [8] introduced the subclass Diaporthomycetidae in the broadest sense with a large number of orders with diverse morphology. To reiterate, the classification of Diaporthomycetidae is changing rapidly, and additional ranks, such as orders and families, have been added to the group. Presently, subclass Diaporthomycetidae includes 21 orders and 66 families that are widespread worldwide and can be found across a wide variety of ecological niches [32]. Hyde et al. [32] provided phylogenetic and divergence time estimations for the subclass and introduced five new orders and six families, which mainly comprise freshwater taxa.

The higher-level taxonomy of Diaporthomycetidae is not yet stable, and this is related to the limited availability of relevant collection and sequence data. Since the 1990s, SSU and LSU have been the most commonly used gene regions for phylogenetic studies of this group [13]. However, these regions could not fulfil species and generic discrimination roles as the data do not feature high variation between the taxa in the subclass. Furthermore, many genera in Diaporthomycetidae have uncertain placements and cannot be assigned to any families or orders. This is mainly because these genera lack sequence data, with many missing lineages. Our new genus is placed in a monophyletic lineage in combined loci phylogeny, with high branch-length support; however, the bootstrap support is low. This may be indicative of more undiscovered diversity in this often-overlooked group. Further, there are no stable and clear conclusions about the boundaries of the higher-level ranks in Diaporthomycetidae. For example, we observed that the family placement in Atractosporales varies on a study-by-study basis [32,33,34]. In the present study, the familiar placement of Pseudoproboscisporaceae is more closely related to Junewangiaceae and not to Atractosporaceae (Figure 2). Therefore, we suggest treating Pseudoproboscisporaceae in the Diaporthomycetidae family *incertae sedis*.

Diatrypaceae species are characterized by a cosmopolitan distribution and often inhabit deadwood and the bark of many plant species. Twenty-two genera are accepted in Diatrypaceae [15], and many have polyphyletic origins [38,39,40]. The stroma morphology, organization of perithecia and number of ascospores per ascus are among the notable morphological characteristics used for generic delineation in many taxa in Diatrypaceae. However, many of the genera in Diatrypaceae have overlapping taxonomic features. Senwanna et al. [40] introduced the genus *Allocryptovalsa* for the species characterized by perithecia immersed in host tissue, polysporous asci and allantoid ascospores. The *Allocryptovalsa* clade (Clade A) includes both the species of the *Allocryptovalsa* and the *Eutypella sensu lato* (Figure 2). The genus *Allocryptovalsa* is characterized by polysporous asci, while *Eutypella* is distinct from *Allocryptovalsa* and *Cryptovalsa* in having eight-spored asci. Eight-spore versus more than eight-spore characteristics have been traditionally used to segregate genera in Diatrypaceae. However, many recent studies have used molecular data to show that the polysporous ascus feature in the Diatrypaceae is of less evolutionary significance [38]. Zhu et al. [39] suggest that both *Allocryptovalsa* and the *Eutypella sensu lato* (Figure 1, clade 12 in their study) ought to be treated as a single genus. We concur with Zhu et al. [39] and place our new species in *Allocryptovalsa* as *A. xishuangbanica*, which is characterized by eight-spored asci. Therefore, in future studies, it would be better to treat the entire clade as *Allocryptovalsa* and provide a broad concept to the genus, which includes both eight-spored and polysporous asci.

In the present study, we isolated three saprobic fungi belonging to Sordariomycetes from deadwood found in Sichuan and Yunnan Provinces. Among them, one genus and two species are new to science. We confirmed that different phenotypes distinguished within Sordariomycetes are phylogenetically distinct. Our study hints at the untapped potential of southwest China as a repository of unknown fungi. We believe that future sampling and increased efforts to cultivate these fascinating fungi in less-studied areas, such as southwest China, will provide fresh insight to the question: where are the missing fungi?

## Data Availability

The datasets generated for this study can be found in the NCBI GenBank, MycoBank and TreeBASE.
